# Effects of Treatment with Various Remineralizing Agents on the Microhardness of Demineralized Enamel Surface

**DOI:** 10.15171/joddd.2015.043

**Published:** 2015-12-30

**Authors:** Kiana Salehzadeh Esfahani, Romina Mazaheri, Leila Pishevar

**Affiliations:** ^1^Post-graduate Student, Department of Pediatric Dentistry, Faculty of Dentistry, Isfahan (Khorasgan) branch, Islamic Azad University, Isfahan, Iran; ^2^Assistant Professor, Department of Pediatric Dentistry, Faculty of Dentistry, Isfahan (Khorasgan) branch, Islamic Azad University, Isfahan, Iran; ^3^Assistant Professor, Department of Operative Dentistry, Faculty of Dentistry, Isfahan (Khorasgan) branch, Islamic Azad University, Isfahan, Iran

**Keywords:** CPP-ACP, demineralization, fluoride varnish, microhardness, remineralization

## Abstract

***Background and aims.*** Remineralization of incipient caries is one of the goals in dental health care. The present study aimed at comparing the effects of casein phosphopeptide-amorphous calcium phosphate complex (CPP-ACP), Remin Pro^®^, and 5% sodium fluoride varnish on remineralization of enamel lesions.

***Materials and methods.*** In this in vitro study, 60 enamel samples were randomly allocated to six groups of 10. After four days of immersion in demineralizing solution, microhardness of all samples was measured. Afterward, groups 1-3 underwent one-time treatment with fluoride varnish, CPP-ACP, and Remin Pro^®^, respectively. Microhardness of groups 4-6 was measured not only after one-month treatment with the above-mentioned materials (for eight hours a day), but also after re-exposing to the demineralizing solution. The results were analyzed by one-way analysis of variance (ANOVA), repeated measures ANOVA, and Fisher’s least significant difference (LSD) test.

***Results***. None of the regimens could increase microhardness in groups 1-3. However, one-month treatment regimens in groups 4-6 caused a significant increase in microhardness. The greatest microhardness was detected in the group treated with CPP-ACP (P = 0.001). In addition, although microhardness reduced following re-demineralization in all three groups, the mean reduction was minimum in the CPP-ACP-treated group (P < 0.001).

***Conclusion. ***While long-term repeated application of all compounds improved microhardness, the remineralization potential of CPP-ACP was significantly higher than that of Remin Pro^®^ and sodium fluoride varnish.

## Introduction

Dental caries prevention and arresting carious lesions are among the main objectives of dental health care. Tooth structure is subject to constant demineralization and remineralization processes in the oral cavity. Therefore, any disturbance in the balance of the oral cavity will destruct tooth structure.^1^ At pH ≤ 5.5, the reaction between hydrogen ions, produced by bacterial metabolism, and the phosphate group of enamel crystals leads to enamel dissolution/demineralization. This process can be reversed at normal pH and in presence of calcium and phosphorus ions. Incipient enamel lesions can be remineralized, especially using treatments to promote remineralization.^2^

Fluoride is believed to prevent dental caries through several mechanisms inducing reduction of acid production by microorganisms, inhibition of intracellular and extracellular enzymes, and replacement of hydroxide ions in hydroxyapatite with fluoride ions (resulting in acid-resistant fluorapatite crystals).^3^ Nevertheless, the substance may also exert undesirable effects such as fluorosis and toxicity in high doses.^4^ Therefore, efforts to find effective anti-caries compounds with minimal side effects have been intensified.

Milk and its derivatives, e.g. cheese, have long been accepted to have anti-caries characteristics. Such protective effects of cheese can be attributed to the chemical impact of its casein phosphoprotein and calcium content.^5^ Casein phosphopeptide-amorphous calcium phosphate complex (CPP-ACP), on the other hand, averts caries by simultaneous inhibition of demineralization and promotion of remineralization.^1,6-9^In addition to having all the potentials of the most common anti-caries material, i.e. fluoride, CPP-ACP is safe, good-tasting, and well-tolerated. Moreover, unlike fluoride, CPP-ACP can be safely swallowed regardless of dose.^10-12^ Thus, if the efficacy of the mentioned complex in enhancing remineralization and suppressing demineralization of dental hard tissue is proven, it might be a proper alternative to a variety of fluoride combinations. Lata et al^2^ and Panich & Poolthong^13^ reported CPP-ACP to encourage remineralization of enamel lesions through different treatment regimens and measurement methods.In addition, Rahiotis et al. concluded that the presence of CPP-ACP on dentin surface decreased the demineralization and enhanced the remineralization of demineralized lesions.^9^

Remin Pro^®^ (Voco, Cuxhaven, Germany) is a novel product containing fluoride, hydroxyapatite, and xylitol which is claimed to prevent the demineralization and erosion of dental tissues. Remin Pro^®^ facilitates the neutralization of acids available in dental plaques and creates equilibrium in the oral flora content. In addition, the fluoride and hydroxyapatite content of the product reinforce remineralization and strengthen enamel surface.

Considering the limited research on the efficacy of this newly introduced product, the present study aimed at comparing the effects of a CPP-ACP paste (GC Tooth Mousse, GC, Tokyo, Japan), Remin Pro^®^(, and 5% sodium fluoride varnish (DuraShield^®^, Sultan Health Care, Redmond, USA) on remineralization of enamel lesions. As surface microhardness of the enamel is related with its minerals content,^14,15^ this index was measured as a criterion to determine enamel demineralization and remineralization.

## Materials and Methods

This in vitro study was performed on 60 human premolars extracted for orthodontic reasons (not only for study). The teeth were immersed in thymol solution (0.2%) post-extraction and maintained at room temperature for about four months. Premolars were only selected if there were no signs of cavity, restoration, crack, abrasion, or hypoplasia. In order to remove the contaminants, the surface of the teeth was scratched by a surgical scalpel blade (No. 15, Steel Surgico Company, Pakistan) and then cleansed using a brush on a slow-speed handpiece and water. Then, tooth roots were cut by a diamond disc (D&Z, Berlin, Germany) and the samples were kept in distilled water at room temperature.

In the next stage, plates with a 2 × 2 mm^2^ square hole in the center were placed at the bottom of special plastic tubes (diameter: 2.5 cm). An epoxy adhesive was then used to mount each tooth crown in one tube with its buccal surface located in the center of the mentioned square. In order to smooth tooth surfaces and increase the accuracy of microhardness measurements, the enamels were polished with a silicon carbide paper (4000 grit size, USA). After the assessment of enamel surfaces under a microscope at ×1000 magnification, the samples were polished again if necessary and those with exposed dentin were discarded.

The samples were randomly allocated to six groups of 10. Baseline surface microhardness in the center of the polished area was measured using a Vickers Hardness Testing Machine (Shimadzu HMV 2000, Kyoto, Japan) with a test force of 245.2 mN and indentation length of 10 s. Caries-like lesions on the enamels were produced by incubating the samples at 37ºC for four days (96 hours). The demineralizing solution contained 2.2 mM calcium chloride (CaCl_2_), 0.05 mM lactic acid, 0.2 parts per million (ppm) fluoride, and 2.2 mM monosodium phosphate (NaH_2_PO_4_).^2^ The pH of the solution was maintained at 4.5 by adding 50% sodium hydroxide (NaOH). The solution was renewed daily to prevent the accumulation of materials produced by demineralization and the consequent pH change. After 96 hours, the surface of each sample was washed with a syringe containing artificial saliva (2.2 mM CaCl_2_, l0.05 mM lactic acid, 0.2 ppm fluoride, and 2.2 mM NaH_2_PO_4_).^2^ They were then placed in containers containing artificial saliva and their microhardness was re-measured.

Then, all samples were air-dried for 30 s and surface treatments were performed. In the first group, 5% sodium fluoride varnish (DuraShield^®^, Sultan Health Care, USA) was left on the surface of the samples for one minute according to the manufacturer’s instructions. Following the immersion of the samples in artificial saliva for one hour, the remaining fluoride varnish was removed by using a No. 15 scalpel blade. Finally, the sample surface were washed with a syringe containing artificial saliva. A similar procedure was followed for the second and third groups. However, surface treatment was conducted by covering the samples with CPP-ACP (GC Tooth Mousse^®^, GC, Japan) for three minute (according to the manufacturer’s instructions) in group 2 and with Remin Pro® (VOCO, Germany) in group 3.

In group 4, the enamels on the dried samples were covered with fluoride varnish. All samples were then placed in special closed containers for eight hours a day. The containers, which contained 1/3 volume artificial saliva, provided a moist environment while preventing contact between the treated surfaces and saliva. Meanwhile, the treated surfaces were moistened with a drop of artificial saliva every four hours. Similar to the first group, the extra varnish was removed from the treated surfaces. The samples were finally washed and immersed in artificial saliva for 16 hours. The same routine was repeated every day for one month. The above-mentioned steps were also applied to groups 5 and 6. However, the surfaces were treated with CPP-ACP and Remin Pro^®^, respectively. In addition, the samples’ surfaces were cleaned with cotton swabs instead of the scalpel blade.

After surface treatments, microhardness of all samples was reassessed. In order to determine the demineralization resistance of the samples, microhardness tests were repeated after were immersing the samples of groups 4-6 in the demineralizing solution for four days.

The mean surface microhardness of all samples was recorded in Vickers hardness number (VHN) in the prepared forms. One-way analysis of variance (ANOVA) was applied to compare the mean surface microhardness values at baseline, after primary demineralization, after treatment regimens, and after re-demineralization. Intragroup comparisons of the mean surface microhardness values at different time intervals were performed using repeated measures ANOVA. In addition, Fisher’s least significant difference (LSD) method was used for pairwise comparisons of the groups in terms of the mean surface microhardness. All analyses were conducted in SPSS for Windows 18.0 (SPSS Inc., Chicago, IL, USA).

## Results

The mean of surface microhardness values in groups 1-3 are presented in Table 1. One-way ANOVA results indicated the absence of significant differences in the mean baseline microhardness (P = 0.260), the mean microhardness after four days of demineralization (P = 0.330), and the mean microhardness after the first treatment (P = 0.120) between groups 1 to 3. On the other hand, pairwise comparisons based on LSD showed that in all three groups, the mean microhardness values after four days of demineralization and after the first treatment were significantly lower than baseline microhardness values (P < 0.001). However, the mean microhardness values after four days of demineralization and after the first treatment were not significantly different in groups 1 (P = 0.780), 2 (P = 0.430), or 3 (P = 0.820).

**Table 1:** The mean surface microhardness values in groups 1-3 treated with fluoride varnish, casein phosphopeptide-amorphous calcium phosphate complex, and Remin Pro^®^

**Groups**	**Microhardness (Vickers hardness number)**		
	**Baseline**	**After four-day demineralization**	**After the first treatment**
**Group 1 (fluoride varnish)**	3.352 ± 5.66	8.230 ± 7.18	9.229 ± 3.19
**Group 2 (CPP-ACP)**	7.344 ± 8.59	4.214 ± 4.44	2.223 ± 7.20
**Group 3 (Remin Pro)**	6.312 ± 8.21	4.210 ± 5.24	1.211 ± 4.17
Values are presented as mean ± SD.			

The mean surface microhardness values in groups 4-6 are summarized in Table 2. According to the results of one-way ANOVA, the three groups had no significant differences in the mean microhardness values at baseline (P = 0.890) and after four days of demineralization (P = 0.410). However, the mean surface microhardness values of the three groups were significantly different after the one-month treatment and after re-demineralization (P < 0.001). Treatment with CPP-ACP paste resulted in the highest microhardness values after both one-month treatment and re-demineralization. The lowest rates, on the other hand, were observed following the application of fluoride varnish. Furthermore, after both the one-month treatment and re-demineralization, the mean microhardness values were significantly higher in group 5 than in group 6 (P < 0.001). Likewise, after the one-month treatment, the mean microhardness was significantly higher in group 6 than in group 4 (P < 0.001). In contrast, no significant difference was observed between groups 4 and 6 after re-demineralization (P = 0.220).

**Table 2:** The mean surface microhardness values in groups 4-6 treated with fluoride varnish, casein phosphopeptide-amorphous calcium phosphate complex, and Remin Pro^®^

**Groups**	**Primary microhardness**		**Microhardness after 4 days demineralization**		**Microhardness after 1 month treatment**		**Microhardness after re-demineralization**	
	**M** ^*^	**SD** ^**^	**M**	**SD**	**M**	**SD**	**M**	**SD**
**Group 1 (fluoride varnish)**	7.339	5.60	6.223	5.39	7.273	3.25	3.267	02.39
**Group 2 (CPP-ACP)**	6.331	05.31	3.235	5.27	9.411	9.35	2.334	09.38
**Group 3 (Remin Pro)**	8.337	02.69	8.230	1.45	5.333	2.25	2.289	1.37
^*^Mean, ^**^standard deviation								

Repeated measures ANOVA suggested that in group 4, the mean microhardness after four days of demineralization was significantly lower than the mean baseline microhardness (P < 0.001). Moreover, the mean microhardness after the one-month treatment significantly increased compared to the microhardness after four days of primary demineralization (P = 0.002). However, there was no significant difference between the mean microhardness values after four days of re-demineralization and after the one-month treatment (P = 0.740). In addition, the mean microhardness after re-demineralization was significantly higher than that after primary demineralization (P = 0.007). While similar findings were observed in groups 5, the mean microhardness value after re-demineralization was significantly lower than that after the one-month treatment (P = 0.002). In group 6, the mean microhardness of the samples significantly decreased after four days of primary demineralization (P < 0.001). On the contrary, the mean value after the one-month treatment was significantly higher than the baseline microhardness (P < 0.001). The mean microhardness value after re-demineralization was significantly lower than that after the one-month treatment (P < 0.001), but still significantly higher than the obtained value after the primary demineralization (P = 0.003).

## Discussion

The prevention of dental caries is more critical than its treatment. Dealing with rapid and progressive caries in infants and young children, called early childhood caries, is a challenge to pediatric dentistry as it usually requires treatment under general anesthesia. Moreover, hormonal changes, adaptation problems, and extreme use of carbohydrates, e.g. sweets and snacks, increase the risk of caries in adolescents.^16^

Dental caries prevention strategies in children and adolescents mainly aim to stop carries in its initial stages and to remineralize the damaged dental surface. In fact, remineralization of the primary decay and white lesions of enamels with preventive materials will in turn decelerate or prevent cavity development and preserve the dental tissue. Specifically-designed treatment regimens have been proven to remineralize initial enamel lesions and increase their acid resistance.^16^ Considering the relationship between mineral content and surface microhardness of dental enamels, the latter can be used as a criterion to assess the efficacy of various remineralizing materials in stopping the demineralization process and reversing lesions. Accordingly, similar to previous research,^2,17^ the mean surface microhardness of all samples at baseline and after the first demineralization was about 330 and 220 VHN, respectively. In other words, although enamels with primary lesions manifested an even surface, their mineral content, and thus microhardness, was lower compared to an intact enamel.

In the present study, we treated artificial caries in groups 1-3 with fluoride varnish for one minute, CPP-ACP for three minutes, and Remin Pro^®^ for three minutes, respectively. One-time professional application of these materials failed to cause remineralization or increased enamel microhardness. Moreover, despite the slight increase in microhardness following the application of CPP-ACP, the three regimens had no significant difference in this regard. Since insufficient exposure time could have been responsible for this finding, repeated application of the mentioned substances over shorter intervals might lead to more favorable results.

Also in this study, groups 4-6 were treated with fluoride varnish, CPP-ACP, and Remin Pro^®^ respectively for eight hours a day over a one-month period. We designed these regimens to mimic the use of the mentioned materials during night at home. Children, who are at high risk of caries development, are instructed to prepare the materials in special trays and put them in their mouth over night after tooth brushing. As the sleeping period in children is usually eight hours on average, selecting the above-mentioned regimens seems logical. Our findings indicated that all three treatment regimens significantly promoted the remineralization of enamel lesions and increased enamel microhardness. Numerous studies have reported similar results in spite of using different treatment regimens and measurement methods.^2,9,13,18-22^

The mechanism of action of these materials can be briefed as follows:

Presence of fluoride ions in the oral cavity causes the precipitation of fluorapatite from existing calcium and phosphate ions in the saliva. Increased pH will then lead to the formation of larger acid-resistant crystals containing fluoride (fluorohydroxyapatite). Consequently, remineralization will be promoted, a strong surface layer will develop, and finally, the resistance of the dental structure to demineralization will be enhanced.^2^

On the other hand, casein phosphate present in CPP-ACP stabilizes calcium and phosphate and facilitates the formation of calcium phosphate nanocomplexes on the tooth surface. These compounds will, in turn, act as a source of minerals for the remineralization process. In fact, the insoluble calcium phosphate becomes soluble in presence of casein phospholipids. Subsequently, amorphous calcium phosphate is formed, localized on the tooth surface, and act as a source of calcium and phosphate ions. It helps calcium and phosphate ions to travel deep into lesions through the porous layer on the white lesion and to encourage the remineralization of enamel crystals. This material is also capable of rapidly turning into hydroxyapatite which is then deposited on the tooth surface.^9,23,24^

Fluoride, hydroxyapatite, and xylitol contents of Remin Pro^®^ reinforce remineralization and strengthen the enamel surface. Since this product significantly increased microhardness (even in comparison to fluoride varnish), its application is recommended not only in conservative treatments, but also after dental bleaching and during orthodontic treatments. According to the manufacturer, Remin Pro^®^ prevents tooth sensitivity by creating a protective layer on the teeth and inhibits bacterial plaque adhesion through smoothing the tooth surface.

While all of the three mentioned materials could substantially increase microhardness of the enamel lesions, CPP-ACP had significantly better outcomes compared to Remin Pro^®^ and fluoride varnish. Likewise, Kumar et al^25^ reported CPP-ACP to be more effective than fluoride in reducing lesion depth. This finding can be attributed to very fine particles of amorphous calcium phosphate (nanocomplexes) present in CPP-ACP, i.e. calcium and phosphate ions can readily diffuse into the porous lesion and penetrate deep into the demineralized lesion. As a result of long-term treatment and adequate exposure time, apatite crystals will form again even in deeper parts. Although the fluoride ion causes an increase in tooth surface resistance to demineralization by deposition of fluorohydroxyapatite, the resulting remineralization, despite its advantages, is a self-limiting phenomenon which prevents penetration of calcium and phosphate ions into deep lesions.^2,3^ The above-mentioned factors may elucidate the higher effectiveness of CPP-ACP in increasing microhardness compared to fluoride varnish and Remin Pro^®^.

Panich & Poolthong^13^ reported that the beneficial effects of CPP-ACP on microhardness of demineralized enamel increased in the presence of artificial saliva (it had a synergistic effect). In other words, immersion of the CPP-ACP-covered samples in artificial saliva, instead of distilled water, substantially enhanced enamel microhardness. The manufacturer has also claimed that longer exposure to the oral environment (i.e. saliva) would increase the efficacy of the product.^13^ We also tried to simulate the oral environment and saliva and found the measured values following the use of CPP-ACP (411.9 VHN) to be even higher than the rates in intact enamel.

In contrast, some researchers have shown the effects of fluoride (in the form of varnish or toothpastes containing fluoride) on remineralization to be similar to or greater than those of CPP-ACP. This inconsistency can be justified by either short-term treatments, i.e. insufficient time for the penetration of materials into deep lesions (especially in the case of CPP-ACP), or the use of distilled water instead of artificial saliva.^2,18,21^

In the present study, groups 4-6 were re-immersed in the demineralizing solution for four days to compare the resistance rate of the samples to demineralization. The results of microhardness tests after this stage revealed that although microhardness was reduced in all three groups, the mean microhardness of the CPP-ACP-treated group was considerably higher than those of the other groups. On the other hand, the minimum difference between microhardness values before and after re-demineralization was detected in the group treated with fluoride varnish. Higher acid resistance of fluorohydroxyapatite compared to hydroxyapatite might have been responsible for this finding. Moreover, resin compounds present in the fluoride varnish, which are capable of penetrating into porous surfaces and inter-prism spaces, could have increased the adhesion of the material to the tooth surface and enhanced the protective effect of fluoride.

Finally, the mean of microhardness values after demineralization of the treated enamel in groups 4-6 were all significantly higher than the values reported from demineralized intact enamel. This finding can be justified by the cumulative effects of mineral (apatite crystals) deposition following long-term treatment with the mentioned compounds. Some previous studies have also suggested surfaces treated with fluoride and CPP-ACP to be more resistant than the intact enamel.^2,26^

The current study design was limited by the simulated oral cavity conditions. Further studies are warranted to compare the effects DIAGNOdent with the mentioned materials through the measurement of microhardness in subsurface layer of treated lesions. In addition, future studies are recommended to measure the depth of lesions under a polarized light microscope before and after treatments.

## Conclusion

1. In the case of repeated and long-term application, all experimental materials (fluoride varnish, CPP-ACP, and Remin Pro^®^) increase remineralization and microhardness of early enamel lesions.
2. Under similar conditions, CPP-ACP causes a significant increase in remineralization of early enamel lesions compared to fluoride varnish and Remin Pro^®^.
3. After re-demineralization, the microhardness of the enamel treated with CPP-ACP is significantly higher than those treated with other materials.
4. Because of the cumulative effects of mineral deposition, after long-term treatment of early enamel lesions with all three mentioned materials, the resistance of tooth surfaces to demineralization is higher than that of intact enamel.

## Acknowledgments

The authors would like to express their deep appreciation to the personnel of metalorgy laboratory of material department of Isfahan University of Technology and the personnel of the laboratory of pharmacology department of Isfahan University of Medical Sciences for their sincere cooperation and their would also like to acknowledge the statistical advisor; and also to dr.seyed ebrahim jabbarifar for his valuabe consultation.

## References

[1] Yamaguchi K, ‏ Miazaki M, Takamizava T, ‏ Inage H, ‏ Moore BK (2006). ‏ Effect of CPP-ACP paste on mechanical properties of bovine enamel as determined by an ultrasonic device. J Dent.

[2] Lata S, Varghese NO, Varughese JM (2010). Remineralization potential of fluoride and amorphous calcium phosphate-casein phosphor peptide on enamel lesions: an in vitro comparative evaluation. J Conserv Dent.

[3] Ten Cate JM (1990). In vitro studies on the effects of fluoride and de- and remineralization. J Dent Res.

[4] Roberson Roberson, Theodore M (2006). Sturdevant’s Art and Science of Operative Dentistry, 5th ed.

[5] White JM, Eakle WS (2000). Rationale and treatment approach in minimally invasive dentistry. J Am Dent Assoc.

[6] Oshiro M, Yamaguchi k, Takamizawa T, Inage H, Watanabe T, Irokawa A, Ando S, Miyzaki M (2007). Effect of CPP-ACP paste on tooth mineralization: an FE-SEM study. J Oral Sci.

[7] Reynolds EC (1997). Remineralization of enamel subsurface lesion by casein phosphopeptide-Stabilized calcium phosphate solutions. J Dent Res.

[8] Ramalingam L, Messer LB, Reynolds EC (2005). Adding casein phosphopeptide-amorphous calcium phosphate to sports drinks to eliminate in vitro erosion. Pediatr Dent.

[9] Rahiotis C, Vougiouklakis G (2007). Effect of a CPP-ACP agent on the demineralization and remineralization of dentin in vitro. J Dent.

[10] Rose RK (2000). Effect of an anticariogenic casein phosphopeptide on calcium diffusion in streptococcal model dental plaques. Arch Oral Biol.

[11] Rose RK (2000). Binding characteristic of streptococcus mutans for calcium and casein phosphopeptide. Caries Res.

[12] Azarpajhooh A, Limeback H (2008). Clincal efficacy of casein derivative: a systematic review of the literature. J Am Dent Assoc.

[13] Panich M, Poolthong S (2009). The effect of casein phosphopeptide-amorphous calcium phosphate and a cola soft drink on in vitro enamel hardness. J Am Dent Assoc.

[14] Featherstone JD, Ten Cate JM, Shariati M, Arends J (1983). Comparison of artificial caries like lesions by quantitative micriradiogarphy and micrihardness profiles. Caries Res.

[15] Gerrard WA, Winter PJ (1986). Evaluation of toothpastes by their ability to assist rehardening of enamel in vitro. Caries Res.

[16] Mc Donald RE, ‏ Avery DR, ‏ Stooky GK, ‏ Chin JR, Kowolik JK (2011). Dental caries in child and adolescent. In: Dean JA,‏ Mc Donald RE,‏ Avery DR, eds. Dentistry for the Child and Adolescent, 9th ed..

[17] Jabbarifar SE, Salavati S, Akhavan A, Khosravi K, Tavakoli N, Nilchian F (2011). Effect of fluoridated dentifrices on surface microhardness of enamel of deciduous teeth. Dent Res J.

[18] Pulido MT, Wefel JS, Hernandez MM, Denehy GE, Guzman-Armstrong S, Chalmers JM, Qian F (2008). The inhibitory effect of MI paste, fluoride and a combination of both on the progression of artificial caries like lesions in enamel. Oper Dent.

[19] Srinivasan N, Kavitha M, Loganathan SC (2010). Comparison of the remineralization potential of CPP-ACP and CPP-ACP with 900 ppm fluoride on eroded human enamel: an in situ 9 study Arch Oral Biol 2010;55:541-4. an in situ 9 study Arch Oral Biol.

[20] Hegde MN, Moany A (2012). Remineralization of enamel subsurface lesions with casein phosphopeptide-amorphous calcium phosphate: a quantitative energy dispersive X-ray analysis using scanning electron microscopy: an in vitro study. J Conserv Dent.

[21] Kim MJ, Lee SH, Lee NY, Lee IH. Evaluation of the effect of PVA tape supplemented with 2.26% fluoride on enamel demineralization using microhardness assessment and scanning electron microscopy: in vitro study. Arch Oral Biol 2012;23. 10.1016/j.archoralbio.2012.06.015 22831864

[22] Uysal T, Amasyali M, Koyuturk AE, Ozcan S (2010). Effect of differences topical agents on enamel demineralizations around orthodontics brackets an in vivo/and in vitro study. Aust Dent J.

[23] Reynolds EC (2008). Calcium phosphate-based remineralization systems: scientific evidence?. Aust Dent J.

[24] Ranjitkar S, Rodriquez JM, Kaidonis JA, Richards LC, Bartlett DW (2009). The effect of casein phosphopeptide amorphous calcium phosphate on erosive enamel and dentin wear by toothbrush abrasion. J Dent.

[25] Kumar VL, Itthagarun A, King NM (2008). The effect of casein phosphopeptide-amorphous calcium phosphate on remineralization of artificial caries-like lesions: an in vitro study. Aust Dent J.

[26] Thaveesangpanich P, Itthagarun A, King NM, Wefel JS (2005). The effects of child formula toothpastes on enamel caries using two in vitro pH -cycling models. Int Dent J.

